# The effect of melatonin on quality of sleep in patients with sleep disturbance admitted to post coronary care units: A randomized controlled trial

**DOI:** 10.37796/2211-8039.1123

**Published:** 2021-03-01

**Authors:** Mohammad Zaman Kamkar, Mehran Mahyar, Seyedmahrokh A. Maddah, Homeira Khoddam, Mahnaz Modanloo

**Affiliations:** a Department of Psychiatry, Golestan Research Center of Psychiatry, Golestan University of Medical Sciences, Gorgan, Iran; b Department of Psychiatry, School of Medicine, Golestan University of Medical Sciences, Gorgan, Iran; c Department of Anesthesiology, Faculty of Medicine, Golestan University of Medical Sciences, Gorgan, Iran; d Nursing Research Center, Golestan University of Medical Sciences, Gorgan, Iran

**Keywords:** Melatonin, Coronary Care Units, Sleep Disorders, Heart Diseases

## Abstract

**Background and objectives:**

Patients with cardiovascular disease who required to be admitted in coronary care units (CCU) would have sleep deprivation. During the admission some factors such as continuous ambient light exposure can suppress melatonin release, in consequence sleep deprivation will be occurred and hinder the progress of patients’ treatment. The aim of study was to evaluate the effect of melatonin on the sleep quality of patients admitted to post-CCU.

**Materials and methods:**

This randomized clinical trial was carried out on 110 patients admitted to post-CCU at SayyadeShirazi Hospital, Gorgan, Iran. Patients with a poor sleep quality (Pittsburgh sleep quality index (PSQI) global score>5) were randomly allocated into two intervention and placebo groups. Patients in the intervention group received melatonin (3 mg; 30 minutes before bedtime), and the placebo group received placebo for 2 weeks, and their sleep quality was re-evaluated after the end of intervention. Data were analyzed using paired *t* test, Wilcoxon, and Chi-square tests by SPSS version 21.

**Results:**

The results showed that mean of the patients’ PSQI scores decreased significantly in intervention group (from 14.95 ± 1.48 to 11.65 ± 1.50, P < 0.001), while in placebo group the difference was not significant (from 15.48 ± 1.47 to 15.24 ± 1.71, P = 0.129). Post-intervention score of patients in intervention group was also lower than the placebo group (P < 0.001).

**Conclusion:**

The melatonin can improve the sleep quality of the patients admitted to post-CCU who suffer from sleep disturbance.

## 1. Introduction

Sleep is an important part of human’s life, and a good night sleep is critical for mental and physical health [1], while insufficient sleep can significantly impair the individual’s quality of life and performance [2]. Sleep disorders, defined as perceived or actual alterations in the quantity or quality of sleep are observed in 26% of general population [3] and over 50% of the elderly [4]. In addition to insomnia, there are other types of sleep disturbances, such as hypersomnia, sleep-related breathing disorders, circadian rhythm sleep disorders, and parasomnias [5].

Several factors are associated with sleep disturbances, such as alcohol, caffeine, and nicotine use [6], mental disorders such as depression and anxiety [7], and physical diseases such as diabetes, obesity, myocardial infarction (MI), stroke, and coronary artery disease [8]. These factors can directly cause sleep disorder by the inconvenience they cause during sleep time, or indirectly by medications used for the disease control. Hospital admission is another factor, which can increase the risk of sleep disturbances [9]. Accordingly, patients admitted to coronary care unit (CCU) are reported to have a poor quality of sleep, because of the underlying cardiac condition, effect of medications, hospital conditions, and stress and anxiety that they experience [10–11]. Considering that poor sleep quality is stressful, it can stimulate the release of epinephrine and norepinephrine, which consequently can be associated with tachycardia, tachypnea, increased myocardial oxygen requirements, and eventually leads to severe ischemia and MI [12]. Therefore, it is essential to pay attention to the sleep of the patients admitted to CCU and post-CCU.

Pharmacological treatment options for modifying sleep include benzodiazepines and sedating agents (such as anxiolytics, antipsychotics, and antihistamines), which may have severe adverse effects such as cognitive impairment [13]. Therefore, melatonin agonists have been suggested for sleep regulation [14]. Melatonin, one of the main hormones of pineal gland, is secreted in response to variations in the circadian cycle and plays a role in organization of sleep-wake rhythm [15]. Accordingly, intravenous and oral administration of melatonin agonists has been suggested for sleep regulation [16]. Studies have shown that melatonin agonists can improve the quality of sleep in several sleep disorders, associated with depression [17] and autism [18], as well as primary sleep disturbances [19]. Other advantages have been also mentioned for melatonin, such as regulation of blood pressure, immune system, and autonomic cardiovascular system, as well as antioxidant actions [20]. Because of the poor quality of sleep in hospital inpatients, especially those who admitted to CCU [21–22] and lower concentration of melatonin reported in such patients [23], we hypothesized that the use of melatonin can also improve the quality of sleep in patients admitted to post-CCU. The aim of this placebo-controlled randomized clinical study was to evaluate the effect of melatonin on quality of sleep of patients with sleep disturbance admitted to post-CCU.

## 2. Methods

This placebo-controlled randomized clinical trial was carried out on patients who were admitted to post-CCU of Sayyade Shirazi Hospital, Gorgan, Iran. The protocol of the study was approved by the Ethics Committee of Golestan University of Medical Sciences (code: IR.GOUMS.REC.1396.83) and was registered in Iranian Registry of Clinical Trials Database (IRCT2017081010340N18). Before patient recruitment, the researcher explained the design and objectives of the study to the patients in person, and asked them to sign the written informed consent form, if they were willing to participate in this study. Considering the results of the study by Jan and colleagues [24], a statistical power of 80%, and a type I error of 5%, the sample size was calculated at 45 patients in each group, which was extended to 55 patients, considering 20% chance of lost to follow-up.

110 eligible patients who were admitted to the post-CCU during the study period were recruited into the study using convenient sampling method. Inclusion criteria were poor sleep quality (Pittsburgh sleep quality index [PSQI]>5), had no physical or psychological disorder causing insomnia such as chronic pain or mood disorders and not using drug that regulates the sleep-wake cycle. Patients who meet the inclusion criteria were randomized into two intervention and placebo groups in a 1:1 ratio using simple random allocation and four-block allocation method. The placebo group was matched in terms of age, sex, and cause of admission with the intervention group. Then patients in both groups fulfill the questionnaires. The questionnaires were administered twice to each group; once before receiving melatonin or placebo and once two weeks afterward. Data was gathered using questionnaire including demographic characteristics (age, sex, ethnicity, and place of residence), clinical characteristics (cause of admission, duration of hospital admission, and history of admission) and PSQI. The quality of sleep was assessed using the Persian version of PSQI, by measuring seven subscales; subjective sleep quality (C1), sleep latency (C2), sleep efficiency (C3), sleep duration (C4), sleep disturbances (C5), use of sleep medications (C6), and daytime dysfunction (C7) over the last month. The patients’ scores in each subscale ranged from 0 to 3, and sleep disturbances of the patients was categorized to no sleep disturbances (score 0), mild (score 1), moderate (score 2), and severe sleep disturbances (score 3). The sum of the scores in the seven subscales was considered as the global score, ranging from 0 to 21. The reliability of Persian version of PSQI in Iranian population was tested previously in the study by Eliyasianfar and colleagues by Cronbach’s alpha of 0.83 [24]. The researcher gave sufficient explanation to the participants about how to complete the questionnaire.

The patients in intervention group received 3 mg melatonin tablets for 14 days which administered 30 minutes before bedtime, and the placebo group received placebo for 14 days, which contained 3 mg chickpea flour that was identical with melatonin in appearance. The drug and the placebo were produced by Mahya Darou, Iran. The placebo was similar to the drug in terms of size, shape, and color and was placed in sealed pockets, to mask the nurse who gave the drugs to the patients. The researcher who gave the questionnaire to the participants and the analyst were also unaware of the group allocations.

After the intervention, the researcher followed up all the participants each 4 days and visited them in person during their hospital admissions. After discharge, the pills were given to the patients and they were asked to continue taking the drug every night routinely until 14 days after initiation of the intervention, and they were asked to complete the PSQI once again. Any patient who was lost to follow-up or did not use the medication routinely (did not take the medicine two times) was excluded from the study.

### 2.1. Statistical analysis

For presenting the results, we described the frequency of qualitative variables and reported mean and standard deviation (SD) for quantitative variables. Shapiro Wilk test was used to test the normal distribution of the data. Comparison of numeric variables between the groups was performed using *t* test or Mann Whitney U test, whenever the data did not appear to have normal distribution. Patients’ scores before and after the intervention were compared using paired samples *t* test or its non-parametric equivalent, Wilcoxon Signed Rank test. Categorical variables were compared using Chisquare test. For the statistical analysis, the statistical software IBM SPSS Statistics for Windows version 21.0 (IBM Corp. 2012. Armonk, NY: IBM Corp.) was used. P values of 0.05 or less were considered as statistically significant.

## 3. Results

**Fig. 1:**
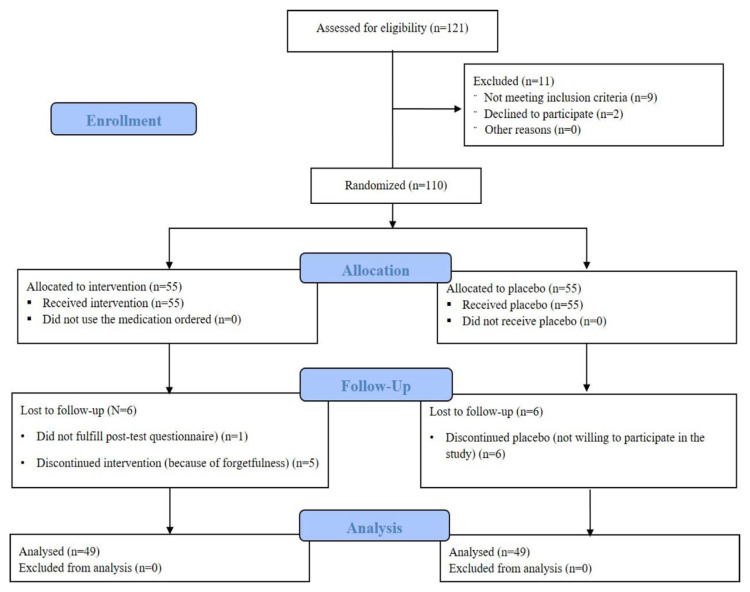
CONSORT flow diagram for study enrollment.

Of the 110 patients enrolled into the study, 98 completed the study (Fig. 1).

There were 30 male patients (61.2%) and 19 female patients (38.8%) in each group. Mean of the patients’ age in the intervention group was 55.04 ± 8.12 years, and that of the placebo group was 56.02 ± 7.08 years (P = 0.53; independent samples *t* test). There was no difference between the groups in terms of ethnicity (P = 0.591), place of residence (P = 0.214), cause of hospital admission (P = 0.817), and admission history (P = 0.417), as shown in Table 1.

Mean of the patients’ PSQI scores decreased significantly in the intervention group from 14.95 ± 1.48 before the intervention to 11.65 ± 1.50 after the intervention (P < 0.001), while the change in the placebo group was not statistically significant (from 15.48 ± 1.47 to 15.24 ± 1.71, P = 0.129). Furthermore, the mean score after the intervention was significantly lower in the intervention group compared with the placebo group (P < 0.001), although the baseline mean scores were not different between the groups (P = 0.079). The mean scores in different subscales of the questionnaire are shown in Table 2. As indicated, the mean baseline scores were similar between the groups (P > 0.05), except in terms of using sleep medications (P < 0.001), while after 2 weeks, the mean scores of the groups were significantly different, lower in the intervention group (P < 0.05; Table 2).

Comparing the change in each group using paired *t* test showed a significant decrease in mean scores of all the seven subscales of PSQI, including subjective sleep quality (P < 0.001), sleep latency (P < 0.001), sleep duration (P = 0.001), habitual sleep efficiency (P = 0.007), sleep disturbances (P < 0.001), using sleep medication (P < 0.001), and daytime dysfunction (P < 0.001) in the intervention group. The change in the mean scores of subjective sleep quality (P < 0.001), sleep duration (P = 0.011), habitual sleep efficiency (P = 0.019), and sleep disturbances (P = 0.044) was also significantly different in the placebo group, indicating decrease in subjective sleep quality and increase in the mean scores of sleep duration, sleep efficiency, and disturbances. However, the change in mean scores of sleep latency (P = 0.133), using sleep medications (P = 0.159) and daytime dysfunction (P = 0.91) was not significant in the placebo group. Studying the association of melatonin with sleep quality based on different variables showed a non-significant effect of age (P = 0.771), sex (P = 0.499), ethnicity (P = 0.821), cause of admission (P = 0.347), and place of residence (P = 0.374).

## 4. Discussion

The present study showed that 14 days administration of 3 mg melatonin, taken once daily, can significantly improve the sleep quality of patients in post-CCU. In our study, the groups were similar regarding mean age, ethnicity, place of residence, cause of hospital admission, and admission history, and the effect of melatonin in the intervention group was not associated with the patients’ demographics or cause of admission. These results show that melatonin could be used for improvement of sleep quality in such patients. It has been previously determined that endogenous melatonin acts as a sleep regulator and its deficiency has been documented in patients suffering from sleep disturbance [25]. Accordingly, exogenous melatonin has been used as a treatment option for regulating sleep and improving the quality of sleep [26]. Measurement of sleep quality using PSQI in breast cancer survivors has shown that 3 mg oral melatonin for 4 months could significantly improve patients’ subjective sleep quality compared with the control group [27], which is similar to the results of the present study, although the type of disease evaluated was different with ours. The effectiveness of melatonin has been also confirmed in patients with other conditions, such as chronic obstructive pulmonary disease [28], depression [17], autism [18], schizophrenia, and Alzheimer’s disease [16], which confirm the results of our study, although the setting and study populations were different.

As patients admitted to CCU have decreased level of melatonin [23] and have an increased risk of poor sleep and impaired quality and quantity of sleep [21–22], it is important to pay attention to their sleep pattern. Accordingly, research has focused on strategies to improve patients’ sleep during their admission. Some researchers have suggested using eye mask and earplugs as easy and cheap methods to be used for improving patients’ sleep in those admitted to intensive care unit (ICU) or CCU [29], while these methods can only control the noise and light effects affecting patients’ sleep and not the pathophysiology of the sleep disturbance [30]. Others have also suggested interventions such as music or aromatherapy to reduce patients’ anxiety and hence, improve their sleep quality in cardiac patients admitted to ICU [31] and CCU [32]. On the other hand, as patients admitted to CCU and post-CCU have underlying cardiac conditions and are taking several medications, it is not safe to prescribe the routine sedatives to these patients, because of the risk of adverse effects, such as respiratory suppression or change in the cardiac rhythm [33], as well as the possible drug interactions between sedatives and other drugs the patients are taking. Therefore, melatonin, which has been confirmed as a safe and efficient sleep regulator, can be an appropriate medication for sleep regulation in critically ill patients [34]. Huang et al. investigated the effect of 3 mg oral melatonin, administered for 4 days, in addition to ear plugs and eye masks in patients admitted to ICU and compared sleep quality and polysomnography results of these patients with placebo. The results showed melatonin as a safe, effective, and easily implementable method for improving sleep quality in these patients [35]. These results have been confirmed in stimulated ICU environment [36–37], and melatonin has shown favorable results in terms of sleep and delirium of patients admitted to ICU [38]. Although the results of these studies confirmed that of ours, regarding the effectiveness of melatonin in critically ill patients, the questionnaire used for evaluating the sleep quality by Huang and partners (Richards Campbell Sleep Questionnaire) [35] was different from that used in our study, PSQI. Furthermore, patients admitted to ICU may have different conditions compared with patients admitted to post-CCU. In another study, Bourne and colleagues investigated the effect of 10 mg oral melatonin compared with placebo, administered for 4 days to critically ill patients receiving mechanical ventilation, using sleep efficiency index (SEI) [39]. The results of this study showed the role of melatonin in increasing nocturnal sleep efficiency and suggested inclusion of melatonin in routine care of critically ill patients [40], which are consistent with the results of the present study, although the selected patients, the administered dose, and duration differed from that of our study. In another study on patients with end stage renal disease undergoing dialysis, 6 weeks administration of 3 mg melatonin compared with a control group, in a cross over design with a 72-hour washout period, showed that melatonin improved total PSQI scores, subjective sleep quality, sleep efficiency, and sleep duration [41], which confirms the results of the present study, although the patients selected were different. Another difference between the results of that study and ours is that we found significant change in all of the seven subscales of PSQI, including subjective sleep quality, sleep latency, sleep duration, habitual sleep efficiency, sleep disturbances, using sleep medication, and daytime dysfunction, and the scores of all subscales of PSQI decreased after intervention in this group; while in the placebo group, the total PSQI score was not different after 2 weeks compared with the baseline. Considering the mean scores of PSQI subscales in the placebo group, the results showed that the mean scores of subjective sleep quality decreased, while the mean scores of sleep duration sleep efficiency, and disturbances increased in this group. Considering the fact that higher scores of PSQI shows worse sleep, the placebo group had not only no improvement in their sleep status in the follow-up period, but also had a worse sleep in most subscales of sleep, including sleep duration, sleep efficiency, and disturbances after 2 weeks, while in the intervention group, mean scores of all of the subscales of PSQI decreased, which shows the effectiveness of melatonin on every subscale of sleep.

Our study have some limitations, including the fact that we enrolled the patients into the study using convenient sampling method and selected patients from one center, which increase the chance of bias and decrease the generalizability of the results. Furthermore, the questionnaire used in the study, PSQI, is a self-report tool and is subject to any bias in the personal views of the participants (subjective bias).

## 5. Conclusion

The results of the present study confirmed the efficacy of 3 mg melatonin, administered for 14 days, in patients with sleep disturbance admitted to post-CCU, which not only improved the global PSQI score, but also decreased the patients’ sleep problem in all of the seven subscales of sleep, measured by this scale. Because of the significance of sleep quality on patients’ health and the prevalence of sleep disturbance in patients admitted to post-CCU, it is suggested to include melatonin in routine care of such patients. Future studies can shed light on the possible adverse effects of melatonin in such patients, and its effectiveness compared with other methods.

## Figures and Tables

**Table 1:** Comparing the demographic and clinical characteristics of patients in the intervention and placebo groups.

Variables		Intervention N (%)	Placebo N (%)	P value^a^
Ethnicity	Fars	31 (63.27)	32 (65.31)	0.591
	Turkmen	11 (22.45)	13 (26.53)	
	Sistani	5 (10.20)	2 (4.08)	
	Baloch	2 (4.08)	1 (2.04)	
	Other	0 (0.0)	1 (2.04)	
Place of residence	Urban	33 (67.3)	27 (55.1)	0.214
	Rural	16 (32.7)	22 (44.9)	
Cause of hospitalization	ACS	37 (75.5)	36 (73.5)	0.817
	MI	12 (24.5)	13 (26.5)	
History of admission	Yes	20 (40.8)	24 (49)	0.417
	No	29 (59.2)	25 (51)	

a The results of Chi-square test.

ACS: Acute coronary syndrome; MI: Myocardial infarction.

**Table 2:** Comparing mean PSQI scores between the groups before and after the intervention.

		Before	After	Mean difference ±SD	P-value^b^
Total score	Intervention	14.95 ± 1.48	11.65 ± 1.50	3.3 ± 1.6	<0.001
	Placebo	15.48 ± 1.47	15.24 ± 1.71	0.24 ± 1.1	0.129
	P-value^a^	0.079	<0.001		
Subjective sleep quality	Intervention	2.20 ± 0.57	0.93 ± 0.62	1.26 ± 0.53	<0.001
	Placebo	2.10 ± 0.58	1.22 ± 0.62	0.87 ± 0.56	<0.001
	P-value^a^	0.387	0.026		
Sleep latency	Intervention	1.87 ± 0.69	1.44 ± 0.50	0.42 ± 0.7	<0.001
	Placebo	1.89 ± 0.62	2.00 ± 0.64	0.1 ± 0.46	0.133
	P-value^a^	0.879	<0.001		
Sleep duration	Intervention	2.02 ± 0.55	1.79 ± 0.53	0.22 ± 0.42	0.001
	Placebo	1.91 ± 0.53	2.12 ± 0.59	0.2 ± 0.53	0.011
	P-value^a^	0.358	0.006		
Habitual sleep efficiency	Intervention	2.42 ± 0.50	2.28 ± 0.50	0.14 ± 0.35	0.007
	Placebo	2.42 ± 0.50	2.59 ± 0.43	0.16 ± 0.47	0.019
	P-value^a^	1.00	0.004		
Sleep disturbances	Intervention	2.26 ± 0.56	2.00 ± 0.57	0.26 ± 0.49	<0.001
	Placebo	2.42 ± 0.50	2.51 ± 0.50	0.08 ± 0.27	0.044
	P-value^a^	0.135	<0.001		
Using sleep medication	Intervention	2.51 ± 0.50	1.81 ± 0.66	0.69 ± 0.68	<0.001
	Placebo	2.91 ± 0.27	2.95 ± 0.19	0.04 ± 0.19	0.159
	P-value^a^	<0.001	<0.001		
Daytime dysfunction	Intervention	1.65 ± 0.69	1.36 ± 0.66	0.28 ± 0.45	<0.001
	Placebo	1.81 ± 0.66	1.80 ± 0.47	0.01 ± 0.2	0.91
	P-value^a^	0.238	0.001		

a P-value based on independent samples *t* test.

b P-value based on paired *t* test.
